# Exploring the role of the immune-neuroendocrine interplay during affective episodes and euthymia in bipolar patients to seek for a reliable biological signature of the disease

**DOI:** 10.1192/j.eurpsy.2024.528

**Published:** 2024-08-27

**Authors:** A. Berry, M. Di Vincenzo, B. Collacchi, L. Giona, A. Giannini, D. D’Amico, F. Cirulli, A. Cirino, M. Luciano

**Affiliations:** ^1^Center for Behavioral Sciences and Mental Health, Istituto Superiore di Sanità, Rome; ^2^Department of Mental and Physical Health and Rehabilitative Medicine, University of Campania “Luigi Vanvitelli”, Naples, Italy

## Abstract

**Introduction:**

Bipolar disorder (BD) is characterised by heterogeneous phenotypic manifestations that may affect the achievement of a timely diagnosis delaying its therapeutic management. Increased circulating levels of pro-inflammatory cytokines and cortisol (CORT) have been observed in BD patients in addition to decreased levels of Brain-Derived-Neurotrophic Factor (BDNF) suggesting that the interaction among these mediators may play a role in the occurrence of affective episodes overall disrupting brain plasticity. However, knowledge on BD etiopathogenesis is still limited, including the causal relationship with inflammatory and neuroendocrine markers.

**Objectives:**

To assess whether variations in peripheral neuroendocrine and inflammatory markers during acute phases of the disease and euthymia might predict the occurrence of affective episodes; to evaluate whether the interplay among these biomarkers might be exploited as a signature of BD.

**Methods:**

We are currently recruiting BD patients during depressive or manic/hypomanic phases together with age- and sex-matched healthy controls (CTRLs). Complete blood count, pro-inflammatory, anti-inflammatory cytokines and BDNF will be assessed in serum; salivary cortisol awakening response test will be used to evaluate hypothalamic-pituitary-adrenal axis activity. MADRS, YMRS and HAM-A will be used to assess psychiatric symptoms, PSP and C-SSRS for global functioning and suicidal risk, IPSS and SRRS for stress levels and CIRS to evaluate physical comorbidities. All assessments will be carried out at the time of recruitment (T0) and after 3 (T1) and 6 (T2) months.

**Results:**

Data have been so far collected on 28 BD patients (18 males, 10 females, age: 48.31±11.3) and 26 CTRLs (16 males, 10 females, age: 46.82±10.86). At T0, BD were characterised by a greater total number of white cells (7.83±1.86 BD vs. 6.78±1.87 CTRL, p<0.05), mean number of neutrophils (4.89±1.49 BD vs. 3.92±1.45 CTRL, p<0.05) and neutrophil/lymphocyte ratio (NLR) (2.52±1.1 BD vs. 1.9±0.69 CTRL, p<0.05). Moreover, BD patients showed overall a greater BMI (30.5±6.6 BD vs. 24.45±3.86 CTRL, p<.001). No difference was observed among groups with respect to sex and age.

**Conclusions:**

Although preliminary, these results suggest that the active phases of BD are associated with a low-grade inflammatory state, potentially related to a different metabolic set-point in BD patients. Ultimately, this study will allow us to evaluate whether the presence of affective symptoms is correlated with fluctuations in the levels of inflammatory mediators, salivary cortisol and BDNF and to establish a reliable and highly predictive BD signature.

**“Funded by: Bando Ricerca Indipendente ISS 2021-2023 to A. Berry project code ISS20-9286e4091f8e”**

**Disclosure of Interest:**

None Declared

